# Severity of imported malaria: protective effect of taking malaria chemoprophylaxis

**DOI:** 10.1186/1475-2875-12-265

**Published:** 2013-07-31

**Authors:** Klaske Vliegenthart-Jongbloed, Mariana de Mendonça Melo, Marlies E van Wolfswinkel, Rob Koelewijn, Jaap J van Hellemond, Perry JJ van Genderen

**Affiliations:** 1 Institute for Tropical Diseases, Harbour Hospital, Haringvliet 72, 3011, TG Rotterdam, The Netherlands; 2 Harbour Hospital and Institute for Tropical Diseases, Laboratory of Parasitology, Haringvliet 72, 3011, TG Rotterdam, The Netherlands; 3 Department of Medical Microbiology and Infectious Diseases, Erasmus Medical Centre & Harbour Hospital, Rotterdam, The Netherlands

**Keywords:** Malaria, Travellers, Prophylaxis, *Plasmodium falciparum*, Travel, Outcome, Import, Severe, Atovaquone, Proguanil, Mefloquine, Immunity

## Abstract

**Background:**

Although chemoprophylaxis remains an important strategy for preventing malaria in travellers, its effectiveness may be compromised by lack of adherence. Inappropriate use of chemoprophylaxis is likely to increase the risk of acquiring malaria, but may probably also worsen the severity of imported cases. The aim of this study was to assess the impact of use of malaria chemoprophylaxis on clinical features and outcome of imported malaria.

**Methods:**

Demographic, clinical and laboratory data of patients included in the Rotterdam Malaria Cohort between 1998 and 2011 were systematically collected and analysed. Patients were classified as self-reported compliant or non-compliant users or as non-users of chemoprophylaxis. Severe malaria was defined using the 2010 WHO criteria.

**Results:**

Details on chemoprophylaxis were available for 559 of the 604 patients, of which 64.6% were non-users, 17.9% were inadequate users and 17.5% reported to be adequate users. The group of non-users was predominated by patients with African ethnicity, partial immunity and people visiting friends and relatives. The majority contracted *Plasmodium falciparum* malaria. In contrast, compliant users acquired non-falciparum malaria more frequently, had significant lower *P. falciparum* loads on admission, shorter duration of hospitalization and significant lower odds for severe malaria as compared with non-users. Patients with *P. falciparum* malaria were more likely to have taken their chemoprophylaxis less compliantly than those infected with non-*P. falciparum* species. Multivariate analysis showed that self-reported adequate prophylaxis and being a partially immune traveller visiting friends and relatives was associated with significantly lower odds ratio of severe malaria. In contrast, age, acquisition of malaria in West-Africa and being a non-immune tourist increased their risk significantly.

**Conclusions:**

Compliant use of malaria chemoprophylaxis was associated with significantly lower odds ratios for severe malaria as compared with non-compliant users and non-users of chemoprophylaxis. After correction for age, gender and immunity, this protective effect of malaria chemoprophylaxis was present only in individuals who adhered compliantly to use of chemoprophylaxis. Patients with *P. falciparum* malaria were more likely to have used their chemoprophylaxis less compliantly than patients with non-*P. falciparum* malaria who were more likely to have contracted malaria in spite of compliant use of chemoprophylaxis.

## Background

Malaria is a mosquito-transmitted disease which may cause a wide variety of symptoms, ranging from no or mild symptoms to severe disease and death. The clinical features and outcome measures of malaria are thought to depend on the infecting *Plasmodium* species, the immune status of the patient, prior use or non-use of chemoprophylaxis, and the timeliness and nature of any treatment administered. Even though the global burden of malaria is largely carried by the world’s malaria-endemic regions with as many as 500 million cases annually and a death toll of one million children each year, malaria may also be acquired by international travellers from non-endemic areas [1]. Every year between 10,000 and 30,000 of these travellers fall ill with malaria after returning home [2]. Estimates indicate that around 150 returning travellers die each year from imported malaria, usually due to *Plasmodium falciparum* infection [1].

Malaria can be prevented through a range of barrier measures to prevent mosquito bites and by taking chemoprophylactic drugs. In areas of intense malaria transmission, malaria chemoprophylaxis remains the most important strategy for preventing malaria in travellers [3], but its use may be associated with adverse outcomes and even death [4]. These potentially severe adverse effects may undermine compliant use of malaria chemoprophylaxis in travellers, in particular when considering that these travellers were usually healthy when commencing travel [5-9]. An additional difficulty with malaria chemoprophylaxis is that all drug regimens must be taken meticulously during and for one or more weeks after leaving the malaria-endemic area [10]. The compliant use of malaria chemoprophylaxis therefore requires considerable personal discipline. Unfortunately, a substantial proportion of travellers may discontinue their anti-malarial drugs soon after returning home because continuation of these drugs after travel may feel counterintuitive [11]. Inappropriate use or early discontinuation of chemoprophylaxis is likely to be a key element in malaria acquisition, but might, speculatively, also worsen the severity of imported cases. In the present study the impact of (self-reported) adherence to malaria chemoprophylaxis on clinical features and outcome of imported malaria was compared to those who reported less compliant use or did not use malaria chemoprophylaxis at all.

## Methods

The Harbour Hospital is a 161-bed general hospital located in Rotterdam, The Netherlands. It also comprises the Institute for Tropical Diseases, which serves as a national referral centre. The Rotterdam Malaria Cohort consists of all patients diagnosed with malaria at the Institute for Tropical Diseases in Rotterdam. In the period 1998–2011 the Rotterdam Malaria Cohort comprised 604 cases of imported malaria. All patients’ demographic, clinical and laboratory data are systematically collected and stored in an electronic database after de-identification. For the present study, data from patients who entered the Rotterdam Malaria Cohort between June 1998 and December 2011 were used to estimate the impact of use of malaria chemoprophylaxis on clinical and laboratory features of imported malaria as well as on the outcome of imported malaria. The outcome measures of interest were: severity of malaria, time in hospital, admission to intensive care unit (ICU), mechanical ventilation, renal replacement therapy, exchange transfusion and death. All available laboratory data were measured on admission with the use of routine procedures.

The standard procedure to diagnose malaria comprised a quantitative buffy coat (QBC) analysis, a rapid diagnostic test (RDT) for malaria antigens Binax NOW® Malaria Test (Binax, Inc, Maine, USA), and thick and thin blood smears using freshly collected blood specimens from finger pricks. The malaria rapid test and the QBC analysis were performed according to the manufacturer’s instructions. QBC capillaries were examined independently by two technicians by microscopic analysis of two complete rows of the region between the bottom of the capillary and the poly-nuclear leukocyte layer using an Olympus BX-60 fluorescence microscope equipped UV-filter and 50x objective and 12.5x oculars (total magnification 625x). Thick blood smears were stained with Field’s stain (Brunschwig Chemie, Amsterdam, The Netherlands) and thin smears were fixed with absolute methanol for three minutes and stained with Diff Quick (Medion Diagnostics, Düdingen, Switzerland). Both staining procedures had been optimized for optimal staining of *Plasmodium* parasites as well as Schüffner’s dots and Maurer’s clefts in infected erythrocytes. Thick and thin smears were examined with regular light microscopes at a total magnification of 1,250x.

### Definitions

#### ***Severe malaria***

Patients were considered as having severe *P. falciparum* malaria if they met the 2010 World Health Organization (WHO) criteria for severe malaria on admission or during hospitalization [12]. These criteria differ from the preset criteria [13] that were used to define severe malaria in previous studies for the Rotterdam Malaria Cohort [14].

### Use of malaria chemoprophylaxis

Compliant use of malaria chemoprophylaxis was defined as self-reported compliant use of malaria chemoprophylaxis, i.e., taken in line with the national guidelines for travellers’ health advice regarding malaria chemoprophylaxis (http://www.lcr.nl). In case the patient did not adhere compliantly to these Dutch guidelines, the use of chemoprophylaxis was classified as non-compliant. Travellers not using any drug for chemoprophylaxis of malaria were labelled as non-users.

### Estimation of immunity to malaria

The degree of immunity to malaria was estimated as follows [15]. Adult immigrants from a malaria-endemic country living in The Netherlands were considered partially immune, because they had likely been exposed to *P. falciparum* during childhood. Immigrant patients who had been born, raised and living in a malaria-endemic area at the time of diagnosis were presumed semi-immune. However, given the relatively low number of semi-immune persons in the Rotterdam Malaria Cohort [15], they were grouped with partially immune individuals. Tourists from non-endemic regions who travelled to endemic areas were considered non-immune.

### Statistical analysis

Data were entered into an Excel database and reviewed for inconsistencies. IBM SPSS statistics 19 (IBM inc, Chicago, IL, USA) was used for statistical analysis, using Chi-square tests for binary outcomes and Kruskal-Wallis analysis to compare continuous variables. Dunn’s post-hoc calculations were performed using GraphPad Instat 3.0 in case of a significant Kruskal-Wallis result. Univariate and multivariate logistic regression was used to assess the impact of several variables on the outcome parameter severe malaria. *P. falciparum* parasite load was not entered as an explanatory variable in the univariate and multivariate analyses of predictors for severe disease, because parasite load is – by definition - one of the defining criteria of severe *P. falciparum* malaria. A *P*-value of less than 0.05 was considered to represent a statistically significant difference.

### Ethical approval

Given its retrospective observational design, ethical approval of this study was not required, according to the Dutch Medical Research Involving Human Subjects Act.

## Results

### Clinical and laboratory features of malaria patients on admission

For 559 of 604 (92.5%) malaria patients included in the Rotterdam Malaria Cohort, details of prophylaxis use were available. The general characteristics of these 559 patients are shown in Table 1. Three hundred and sixty-one (64.6%) malaria patients did not use any form of malaria chemoprophylaxis, 100 (17.9%) used malaria prophylaxis inadequately whereas 98 (17.5%) patients presented with malaria despite adequate use of malaria chemoprophylaxis. Ethnicity differed significantly between the three prophylaxis groups with Caucasians being over-represented in the adequate prophylaxis group. African ethnicity was more common in the group of patients not using malaria chemoprophylaxis. When immunity status towards malaria was estimated, the group of patients without chemoprophylaxis was predominated by patients with presumed partial immunity. The presumed immune status of the majority of adequate users of malaria chemoprophylaxis was non-immune (Table 1). With regard to purpose of travel, tourists comprised a substantial proportion of the adequate users group, whereas the group of non-users was dominated by visiting friends and relatives (VFRs) and sailors. There were no significant differences in seasons of travel. The majority of the non-users contracted malaria in Africa, whereas almost half of the patients with compliant use of chemoprophylaxis contracted malaria outside of Africa. Vital signs on admission did not differ between the three patient groups. Admission C-reactive protein and creatinine levels were significantly higher in non-users as compared with compliant users (Table 1).

**Table 1:** General characteristics of malaria patients entered in the Rotterdam Malaria Cohort grouped according to self-reported adherence with malaria chemoprophylaxis

		**Non-users**	**Non-compliant users**	**Compliant users**	**P-values**
		**n = 361**	**n = 100**	**n = 98**	
**Demographics**					
Age, years		39 (5–70)	37 (13–60)	38 (4–77)	N.S.
Gender, n (%)					N.S.
	Male	263 (73)	73 (73)	62 (63)	
	female	98 (27)	27 (27)	36 (37)	
Ethnicity, n (%)					<0.001
	Caucasian	148 (42)	64 (65)	78 (80)	
	African	160 (45)	31 (31)	14 (14)	
	Asian	32 (22)	3 (5)	3 (3)	
	Other	16 (10)	1 (3)	3 (3)	
Immunity, n (%)					<0.001
	Non immune	138 (49)	50 (64)	30 (81)	
	Partially immune	139 (61)	28 (36)	7 (19)	
**Travel history**					
Reason for travel, n (%)					<0.001
	Tourist	69 (21)	28 (30)	35 (51)	
	VFR	114 (35)	27 (29)	11 (16)	
	Business	54 (17)	27 (29)	15 (22)	
	Expat	25 (8)	4 (4)	3 (4)	
	Sailor	37 (11)	3 (3)	4 (6)	
	Other	24 (7)	4 (4)	1 (1)	
Continent of infection, n (%)					<0.001
	African	290 (82)	82 (82)	54 (58)	
	Asian	42 (12)	10 (10)	22 (24)	
	Americas	19 (5)	7 (7)	14 (15)	
	Other	1 (0)	1 (1)	3 (3)	
Duration of complaints, n (%)					N.S.
	Less than 8 days	228 (63)	70 (70)	46 (47)	
	8–14 days	66 (18)	18 (18)	21 (21)	
	15–21 days	25 (7)	6 (6)	6 (6)	
	>28 days	9 (3)	5 (5)	2 (2)	
	Unknown	34 (9)	1 (1)	23 (24)	
		n = 361	n = 100	n = 98	
**Vital signs**					
Temperature, **°**Celsius		38.5 (35.0–41.5)	38.8 (36.1–40.9)	38.5 (36.0–41.2)	N.S.
Systolic blood pressure, mmHg		120 (64–185)	123 (90–190)	120 (90–196)	N.S.
Pulse rate, beats per minute		92 (50–150)	94 (56–130)	96 (60–150)	N.S.
**Laboratory features**					
Haemoglobin, mmol/L		8.3 (2.5–11.0)	8.3 (4.0–11.1)	8.4 (4.2–10.7)	N.S.
Thrombocytes, x10^9^/L		84 (2–293)	99 (3–302)	96 (19–258)	N.S.
Leucocyte count, x10^9^/L		5.3 (1.3–18.5)	5.1 (1.9–13.4)	5.0 (1.5–12.9)	N.S.
CRP, mg/L		96 (5–476)	77 (7–310)	71 (6–287)	0.0009 ^A: p<0.01, B: p<0.05^
Creatinine, μmol/L		93 (39–1.081)	95 (55–213)	87 (46–405)	0.0019 ^B: p<0.01, C: P<0.01^
LDH, U/L		265 (103–2.297)	270 (98–877)	237 (98–664)	N.S.
Bilirubin, μmol/L		24 (3–416)	22 (6–95)	23 (5–262)	N.S.
Lactate, mmol/L		1.6 (0.5–6.2)	1.6 (0.5–4.7)	1.3 (1.1–1.8)	N.S.

Legend: n = 559. Data are given as median (range) or number (%), as appropriate.

A: No prophylaxis *versus* inadequate prophylaxis, B: no prophylaxis *versus* adequate prophylaxis, C: inadequate prophylaxis *versus* adequate prophylaxis, *N.S.* not statistically significant.

### Outcome of malaria patients

**Figure 1:**
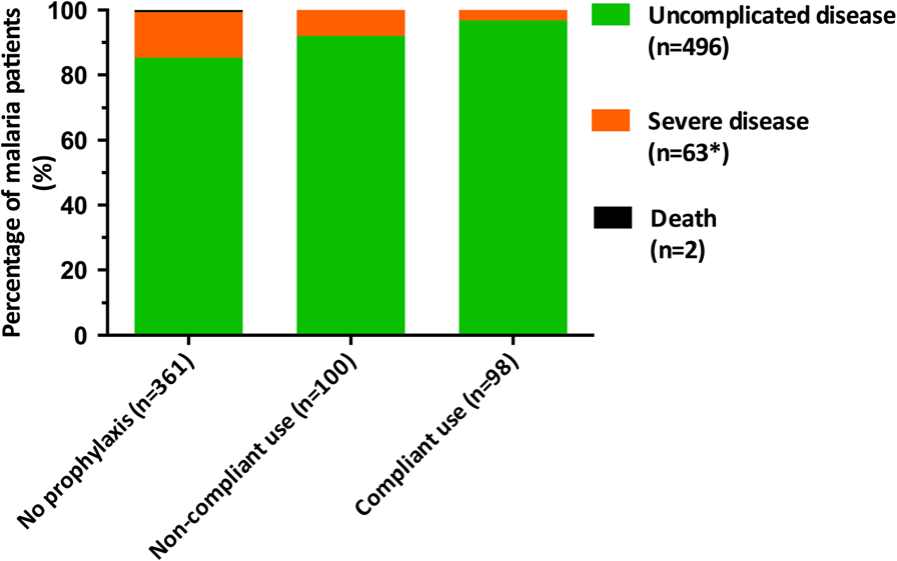
**Outcome of malaria patients in the Rotterdam Malaria Cohort in relation to self-reported use of malaria chemoprophylaxis.** Legend: * = including the two fatal cases.

Patients with compliant use of malaria chemoprophylaxis had significantly lower odds ratios for the outcome parameter severe malaria (OR 0.121 (95% CI 0.029-0.516) than patients not using malaria prophylaxis (Table 2, Figure 1). In addition, patients with compliant use of malaria chemoprophylaxis also had lower odds ratios for admission to ICU and shorter times in hospital (Table 2). Also in univariate and subsequent multivariate analyses, age, region of acquisition, adequate use of malaria chemoprophylaxis as well as non-immune tourists and partially immune VFRs were identified as independent predictors for severe malaria (Table 3). Interestingly, non-immune tourists had significant higher odds ratios whereas partially-immune VFRs had significantly lower odds ratios for severe malaria. The combined variables non-immune tourists and partially immune VFRs were used to diminish the number of variables in the multivariate analyse but also to partially negotiate for the (confounding) interaction between non-immunity and tourists on one hand and partially-immunity and VFRs on the other hand.

**Table 2:** Outcome measures of patients with malaria, grouped according to self-reported adherence with malaria chemoprophylaxis

**Outcome measure**	**Group**	**Proportion or mean (95% CI)**	**Odds ratio (95% CI)**	***P*-value (with no chemoprophylaxis as comparator)**
**Severe malaria**	No chemoprophylaxis	53/361	Reference	
**(WHO 2010)**	Non-compliant use	8/100	0.505 (0.232–1.102)	N.S.
	Compliant use	2/98	0.121 (0.029–0.516)	0.0002
**ICU admission**	No chemoprophylaxis	73/361	Reference	
	Non-compliant use	15/100	0.696 (0.380–1.276)	N.S.
	Compliant use	6/98	0.257 (0.108–0.611)	0.0008
**Mechanical ventilation**	No chemoprophylaxis	3/361	Reference	
	Non-compliant use	1/100	1.205 (0.124–11.722)	N.S.
	Compliant use	0/98	0.520 (0.027–10.158)	N.S.
**Renal replacement therapy**	No chemoprophylaxis	7/361	Reference	
	Non-compliant use	0/100	0.235 (0.013–4.150)	N.S.
	Compliant use	0/98	0.240 (0.014–4.241)	N.S.
**Exchange transfusion**	No chemoprophylaxis	35/361	Reference	
	Non-compliant use	6/100	0.595 (0.243–1.457)	N.S.
	Compliant use	1/98	0.096 (0.013–0.710)	0.0024
**Death**	No chemoprophylaxis	2/361	Reference	
	Non-compliant use	0/100	0.715 (0.034–15.033)	N.S.
	Compliant use	0/98	0.730 (0.035–15.341)	N.S.
***P. falciparum *species**	No chemoprophylaxis	285/358*	Reference	
	Non-compliant use	77/100	0.858 (0.504–1.460)	N.S.
	Compliant use	41/98	0.184 (0.114–0.297)	<0.0001
***P. falciparum *parasite load (asexual parasites per μL)**	No chemoprophylaxis	73,774 (55,620–91,928)	Reference	
	Non-compliant use	48,866 (20,264–77,467)	N.A.	N.S.
	Compliant use	24,563 (5,163–43,963)	N.A.	0.0004
**Time in hospital (days)**	No chemoprophylaxis	4.8 (4.4–5.2)	Reference	
	Non-compliant use	4.8 (4.4–5.3)	N.A.	N.S.
	Compliant use	2.9 (2.4–3.5)	N.A.	<0.0001

Legend: * = 3 patients with a mixed *P. falciparum*/non-falciparum infection were excluded, *CI* confidence interval, *N.A.* not applicable, *N.S.* not statistically significant.

**Table 3:** Univariate and multivariate logistic regression of predictors for severe malaria

***Variables***		**Univariate analysis**		**Multivariate analysis**	
		***P*-value**	**Odds ratio (95% CI)**	***P*-value**	**Odds ratio (95% CI)**
**Age**		0.000	1.048 (1.024–1.071)	0.003	1.037 (1.013–1.062)
**Sexe**	*Male*	0.256	0.726 (0.417–1.262)	0.181	0.661 (0.360–1.213)
	*Female*	0.256	1.378 (0.792–2.398)		
**Immunity**	*Non-immune*	0.000	3.944 (2.249–6.915)		
	*Partially or semi immune*	0.860	0.950 (0.537–1.681)		
**Reason for travel**	*Tourist*	0.196	1.464 (0.822–2.608)		
	*VFR*	0.036	0.470 (0.233–0.950)		
	*Business*	0.676	1.154 (0.590–2.258)		
	*Expat*	0.175	1.903 (0.751–4.819)		
	*Sailor*	0.050	2.195 (1.001–4.812)		
	*Other*	0.450	0.570 (0.132–2.454)		
**Region of acquisition**	*Outside Africa*	0.003	0.288 (0.128–0.647)		
	*North Africa*	0.999	N.A.		
	*West Africa*	0.000	3.567 (1.969–6.462)	0.000	3.287 (1.753–6.161)
	*Central Africa*	0.142	0.409 (0.124–1.350)		
	*East Africa*	0.356	0.639 (0.246–1.656)		
	*Southern Africa*	0.543	1.984 (0.218–19.033)		
**Season of infection**	*March-August*	0.362	0.780 (0.457–1.331)		
	*September-February*	0.362	1.283 (0.751–2.190)		
**Prophylaxis**	*Adequate prophylaxis*	0.006	0.137 (0.033–0.569)	0.007	0.132 (0.031–0.567)
	*Inadequate prophylaxis*	0.257	0,639 (0.294–1.387)	0.143	0.540 (0.236–1.233)
	*No prophylaxis*	0.001	3.235 (1.607–6.512)		
**Duration of illness before diagnosis**	*1 to 7 days*	0.670	0.882 (0.496–1.568)		
	*8 to 14 days*	0.115	1.633 (0.887–3.005)		
	*15 to 28 days*	0.478	0.644 (0.196–2.168)		
	*More than 28 days*	0.478	0.644 (0.196–2.168)		
**Combined variables**	*Non-immune tourists*	0.000	3.274 (1.770–6.058)	0.026	2.200 (1.101–4.396)
	*Partially-immune VFR*	0.059	0.476 (0.221–1.029)	0.045	0.426 (0.185–0.981)

Legend: Included n = 559, *CI* confidence interval, *VFR* visiting friends and relatives, *N.A.* not applicable.

### Causative *Plasmodium* species in relation to use of malaria chemoprophylaxis

**Figure 2:**
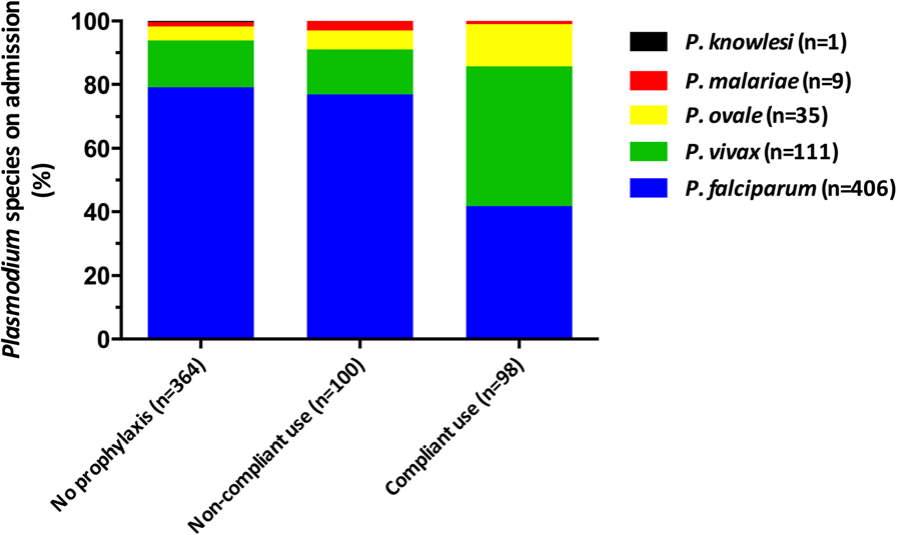
**Distribution of *Plasmodium *species diagnosed in the Rotterdam Malaria Cohort in relation to self-reported use of malaria chemoprophylaxis.** Note that the total number of people without prophylaxis is higher due to three mixed infections.

**Figure 3:**
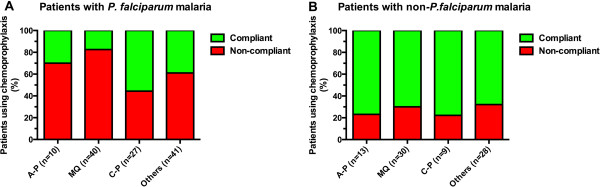
**The proportion of compliant *versus *non-compliant use of specified chemoprophylaxis in patients with malaria. A**: in patients presenting with *P. falciparum* malaria, **B**: in patients presenting with *non-P. falciparum* malaria. Legend: A-P = atovaquone-proguanil; MQ = mefloquine; C-P = chloroquine-proguanil.

The distribution of the causative *Plasmodium* species in relation to the use of and adherence with malaria chemoprophylaxis is shown in Figure 2. The majority of the *Plasmodium* species in malaria patients who did not use malaria chemoprophylaxis or did not compliantly adhere to malaria chemoprophylaxis were identified as *P. falciparum*. In contrast, non-*P. falciparum* species were identified as the causative species of malaria in the majority of malaria patients who used malaria chemoprophylaxis compliantly (Figure 3). In fact, of the group of malaria patients who reported use of malaria chemoprophylaxis (either compliantly or non-compliantly), patients with *P.falciparum* malaria had significantly higher odds ratio for non-compliant use than patients who presented with non-*P. falciparum* malaria (Table 4, Figure 3). At the level of specified drug use, this observation was also valid in atovaquone-proguanil and mefloquine users but not in chloroquine-proguanil users. Conversely, malaria patients who presented with non-*P. falciparum* species were more likely to have contracted malaria in spite of compliant use of chemoprophylaxis (Table 4, Figure 3).

**Table 4:** **Self-reported adherence with and drugs used for malaria chemoprophylaxis in relation to causative *Plasmodium *species**

		**Self-reported adherence to chemoprophylaxis**			**The odds ratio for non-compliant use of prophylaxis in *P. falciparum *infections *versus *non-*P. falciparum *infections**
***Drug used for chemoprophylaxis***	***Malaria species***	***Non compliant (n = 100)***	***Compliant (n = 98)***	***P*-value**	**Odds ratio (95% CI)**
Atovaquone-proguanil	*P. falciparum*	7	3	**0.024**	**7.778 (1.200–50.424)**
	Non-falciparum	3	10		
Mefloquine	*P. falciparum*	33	7	**<0.001**	**11.000 (3.556–34.023)**
	Non-falciparum	9	21		
Chloroquine-proguanil*	*P. falciparum*	12	15	**0.236**	**2.800 (0.489–16.036)**
	Non-falciparum	2	7		
Others	*P. falciparum*	25	16	**0.019**	**3.299 (1.200–9.069)**
	Non-falciparum	9	19		
**Total**	***P. falciparum***	**77**	**41**	**<0.001**	**4.654 (2.517–8.607)**
	**Non-falciparum**	**23**	**57**		

Legend: *N.A.* not applicable, *CI* confidence interval, * = chloroquine-proguanil combination only.

### Causative *Plasmodium* species in relation to outcome severe malaria

Even though the occurrence of severe malaria is not explicitly confined to *P. falciparum* species alone and severe cases of non-*P. falciparum* have been recognized (especially in cases with *P. vivax* and *P. knowlesi* infections), all cases of severe malaria in the Rotterdam Malaria Cohort were associated with *P. falciparum* infections. For this reason, the univariate and multivariate statistical analysis was also done separately for malaria patients with infections solely caused by *P. falciparum* species (Additional file 1). The parameters age, region of acquisition, adequate use of malaria chemoprophylaxis and the combined variable partially-immune VFR were again identified as independent predictors for severe (*P. falciparum*) malaria but the combined variable non-immune tourists lost its statistical significance due to the reduction in number of eligible cases.

## Discussion

Deaths from malaria among travellers are known to be related to delays in treatment and failure to comply with anti-malarial chemoprophylaxis [16]. Both case fatalities in the current study occurred in patients not taking chemoprophylaxis. However, statistical analysis did not reveal a significant difference in case-fatality rates between compliant users, non-compliant users and non-users, which was conceivably due to the very low number of case fatalities in the Rotterdam Malaria Cohort.

Evidence is accumulating that severe *P. falciparum* malaria tends to occur more commonly in patients not taking malaria chemoprophylaxis [17]. This may be particularly true for the non-immune traveller to malaria-endemic regions, especially elderly persons [18,19] as became also evident from the current study. Compliant use of anti-malarial chemoprophylaxis was associated with a significantly lower odds ratio for severe malaria. In addition, compliant users of chemoprophylaxis with malaria had significantly lower *P. falciparum* loads on admission as compared to those who took no chemoprophylaxis at all or did not comply with proper use. This reduced parasite burden in compliant users of chemoprophylaxis probably directly relates to a decrease in risk since many studies documented that high parasite loads are independently associated with worse outcome [18,20]. In terms of outcome, non-compliant users did not significantly differ from non-users. The protective effects of the currently recommended drugs for chemoprophylaxis of malaria was only present when taken as indicated.

Among travellers there is also increasing evidence that severe malaria tends to occur less frequently in people of African origin [21,22]. This reduced risk is commonly attributed to some degree of residual immunity towards malaria. The findings of the present study are in line with this. Individuals with partial immunity visiting friends and relatives had significant lower odds of severe malaria. Conversely, non-immune tourists had significant higher odds for severe malaria and a more complicated course. Even when the protective effects of partial immunity and the risk of non-immunity were taken into account in the multivariate analysis, the protective effect of compliant use of chemoprophylaxis on odds for severe malaria remained present as an independent factor. This suggests that partially immune VFRs may further reduce their risk for severe malaria by strict adherence to anti-malarial chemoprophylaxis. Of note, and in line with other studies [19], increasing age was also in the current study identified as an independent risk factor for a worse outcome. Pre-travel health advice should stress the importance of compliant use of chemoprophylaxis in each traveller but – given their risk profile [23] - particularly in elderly travellers, non-immune tourists and last-minute-travellers [19,23].

In areas of intense malaria transmission, malaria chemoprophylaxis remains the most important strategy for prevention of malaria in non-immune travellers. A recent Cochrane review, however, provided inconclusive evidence about which of the currently recommended drugs atovaquone/proguanil, mefloquine and doxycycline, was most effective in preventing malaria in non-immune populations travelling to regions with *P. falciparum* resistance to chloroquine [24]. Interestingly, occasional failures of anti-malarial chemoprophylaxis have been documented for any chemoprophylactic regimen [25-27]. The current findings of the acquisition of malaria by travellers despite compliant use of malaria chemoprophylaxis underline these potential restrictions. Even though it can not be excluded that resistance patterns towards the commonly used chemoprophylactic drugs might have changed over time during the course of this study, the findings of the current study stress the notion that the occurrence of *P. falciparum* malaria in a person taking malaria chemoprophylaxis was more likely to be associated with non-compliant than with compliant use. This finding was not only demonstrable in the whole group of malaria patients taking chemoprophylactic drugs, but also valid for those individuals taking either atovaquone/proguanil or mefloquine for prevention of malaria, in line with their biochemical mode of action. In contrast, this differential effect was not present in chloroquine-proguanil users, which might raise additional concerns on the efficacy of chloroquine-proguanil for prevention of *P. falciparum* malaria. In contrast, the occurrence of non-*P. falciparum* malaria in a subject taking malaria chemoprophylaxis was more likely to occur in spite of compliant use of a chemoprophylactic drug. This observation is not surprisingly since the labelled indication of chemoprophylactic anti-malarials is to prevent the acquisition of *P. falciparum* malaria only [25-27].

### Limitations

A major limitation of any retrospective analysis is that data were not originally recorded with this type of study in mind. In addition, there is no exact information about the total number of travellers that were at risk of malaria and their compliance with regard to prophylaxis during the study period. This limits the outcomes to odds ratios instead of relative risks. Further, one could argue that estimating the degree of compliance, especially self-reported compliance, may have introduced an important selection and recall bias. Previous studies comparing self-reported compliance with prophylactic anti-malarials and measurements of blood levels showed that patients significantly overestimated their degree of compliance [28]. However, even within the framework of these limitations, the parameter self-reported compliance with malaria chemoprophylaxis should be considered a valuable asset for clinical decision making and risk assessment. With the use of the current straightforward clinical criteria for compliant and non-compliant use of malaria chemoprophylaxis, only compliant use of malaria chemoprophylaxis was identified as an independent predictor in multivariate analysis, significantly reducing the risk of severe malaria. These protective effects remained present after adjustment for the potentially confounding protective effects of partial immunity and being a VFR traveller. Of note, these protective effects of anti-malarial chemoprophylaxis were not demonstrable for non-compliant users.

## Conclusions

Compliant use of malaria chemoprophylaxis was associated with significantly lower odds ratios for the occurrence of severe malaria and admission to ICU. These protective effects of malaria chemoprophylaxis on outcome of malaria were only present when malaria chemoprophylaxis was taken as indicated. Increasing age, acquisition of malaria in West Africa and being a non-immune tourist were identified as independent predictors associated with an increased risk of severe malaria. Partially immune individuals, in particular those visiting friends and relatives had significantly lower odds ratios for severe malaria. These important protective effects of compliant use of malaria chemoprophylaxis should be stressed in future pre-travel health encounters with travellers to tropical regions, especially with presumed non-immune and elderly travellers.

## Competing interests

The authors declare that they have no competing interests.

## Authors’ contributions

PJJvG made the design of the study, MEvW and PJJvG participated in collecting data and planning analysis and evaluation of data. RK prepared and maintained the Rotterdam Malaria Cohort database. KVJ performed cleaning and extraction of data. KVJ and PJJvG performed statistical analysis and participated in literature review. PJJvG and KVJ drafted the first version of the manuscript. All authors gave input and revised, reviewed and approved the final version of the manuscript.

## Supplementary Material

Additional file 1 **Univariate and multivariate logistic regression of predictors for severe *P. falciparum* malaria.** Click here for file
