# Supplementary material for: Severity of imported malaria: protective effect of taking malaria chemoprophylaxis
Source: Malar J. 2013 Jul 31;12:265. doi: 10.1186/1475-2875-12-265 (PMC3734097; doi:10.1186/1475-2875-12-265)
Supplement: Additional file 1 — Univariate and multivariate logistic regression of predictors for severe P. falciparum malaria. [file 1475-2875-12-265-S1.docx]

**Additional file 1:** Univariate and multivariate logistic regression of predictors for severe *P. falciparum* malaria

| ***Variables*** | | **Univariate analysis** | | **Multivariate analysis** | |
| --- | --- | --- | --- | --- | --- |
|  |  | ***P*-value** | **Odds ratio (95% CI)** | ***P*-value** | **Odds ratio (95% CI)** |
| **Age** |  | 0.000 | 1.050 (1.024-1.075) | 0.006 | 1.035 (1.010-1.060) |
| **Sexe** | *Male* | 0.130 | 0.643 (0.363-1.139) | 0.139 | 0.626 (0.336-1.164) |
|  | *Female* | 0.130 | 1.555 (0.878-2.755) |  |  |
| **Immunity** | *Non-immune* | 0.007 | 2.188 (1.236-3.873) |  |  |
|  | *Partially or semi immune* | 0.047 | 0.556 (0.311-0.991) |  |  |
| **Reason for travel** | *Tourist* | 0.046 | 1.847 (1.012-3.372) |  |  |
|  | *Visiting Friends and Relatives (VFR)* | 0.002 | 0.317 (0.156-0.645) |  |  |
|  | *Business* | 0.810 | 1.088 (0.547-2.162) |  |  |
|  | *Expat* | 0.276 | 1.700 (0.654-4.417) |  |  |
|  | *Sailor* | 0.021 | 2.692 (1.165-6.221) |  |  |
|  | *Other* | 0.600 | 0.670 (0.150-2.989) |  |  |
| **Region of acquisition** | *Outside Africa* | 0.706 | 0.849 (0.364-1.982) |  |  |
|  | *North Africa* | 0.999 | N.A. |  |  |
|  | *West Africa* | 0.045 | 1.860 (1.013-3.415) | 0.048 | 1.907 (1.004-3.620) |
|  | *Central Africa* | 0.071 | 0.331 (0.100-1.101) |  |  |
|  | *East Africa* | 0.537 | 0.735 (0.277-1.952) |  |  |
|  | *Southern Africa* | 0.781 | 1.367 (0.150-12.435) |  |  |
| **Season of infection** | *March-August* | 0.329 | 0.761 (0.440-1.316) |  |  |
|  | *September-February* | 0.329 | 1.313 (0.760-2.271) |  |  |
| **Prophylaxis** | *Adequate prophylaxis* | 0.065 | 0.256 (0.060-1.087) | 0.025 | 0.184 (0.042-0.811) |
|  | *Inadequate prophylaxis* | 0.172 | 0.578 (0.263-1.269) | 0.158 | 0.550 (0.240-1.262) |
|  | *No prophylaxis* | 0.014 | 2.436 (1.194-4.970) |  |  |
| **Duration of illness before diagnosis** | *1 to 7 days* | 0.340 | 0.749 (0.414-1.355) |  |  |
|  | *8 to 14 days* | 0.049 | 1.886 (1.002-3.553) |  |  |
|  | *15 to 28 days* | 0.500 | 0.654 (0.190-2.246) |  |  |
|  | *More than 28 days* | 0.500 | 0.654 (0.190-2.246) |  |  |
| **Combined variables** | *Non-immune tourists* | 0.011 | 2.238 (1.203-4.166) | 0.214 | 1.552 (0.775-3.106) |
|  | *Partially-immune VFR* | 0.001 | 0.285 (0.131-0.618) | 0.007 | 0.323 (0.141-0.739) |

Legend: included n= 406; CI = confidence interval; VFR = visiting friends and relatives; N.A.= not applicable
